# Evaluation of the Protective Effects of Doxycycline on Acetaminophen-Induced Hepatotoxicity in Mice

**DOI:** 10.22037/ijpr.2019.1100669

**Published:** 2019

**Authors:** Shirin Soltani, Mohammad Javad Khodayar, Hamid Yaghooti, Maryam Salehcheh, Esrafil Mansouri, Leila Zeidooni, Fereshteh Dehbashi, Azin Samimi

**Affiliations:** a *Faculty of Allied Pharmacy, Ahvaz Jundishapur University of Medical Sciences, Ahvaz, Iran. *; b *Toxicology Research Center, Ahvaz Jundishapur University of Medical Sciences, Ahvaz, Iran.*; c *Department of Toxicology, School of Pharmacy, Ahvaz Jundishapur University of Medical Sciences, Ahvaz, Iran.*; d *Department of Medical Laboratory Sciences, School of Allied Medical, Ahvaz Jundishapur University of Medical Sciences, Ahvaz, Iran. *; e *Cellular and Molecular Research Center, Department of Anatomical Sciences, School of Medicine, Ahvaz Jundishapur University of Medical Sciences, Ahvaz, Iran.*

**Keywords:** Acetaminophen, Liver injury, Oxidative stress, Doxycycline, Hepatoprotective, Mice

## Abstract

Acetaminophen (APAP) toxicity threatens human health due to increased mortality associated with its overdose. Doxycycline (DC) because of its properties such as antioxidant and anti-inflammatory can be a good therapeutic strategy to treat the acute toxicity induced by APAP. Male mice were divided into six groups in two periods of 3 h and 24 h as normal saline, APAP 400 mg/kg, DC 100 mg/kg and groups treated by 25, 50 and 100 mg/kg DC just before APAP, respectively. At the end of the 3 h and 24 h periods, the hepatic index, biochemical parameters including serum aspartate transaminase (AST) and alanine transaminase (ALT) activity and hepatic catalase activity, glutathione (GSH) and malondialdehyde (MDA) levels in liver and histopathological changes were evaluated. The results indicated that DC had no apparent effect on the hepatic index but significantly normalized the level of biochemical parameters and reduced APAP induced liver damage. Overall, it could be concluded that DC can inhibit or resolve harmful effects of APAP through antioxidant and anti-inflammatory properties. However, more studies are needed to understand exact mechanism of DC and its application for clinical use.

## Introduction

Acetaminophen (APAP) or N-acetyl-para-aminophenol is commonly used as an analgesic and antipyretic drug (1, 2). At the therapeutic doses, APAP is primarily metabolized by glucuronidation and sulfation. However, during APAP overdose, excessive N-acetyl-p-benzoquinone imine (NAPQI) as a toxic metabolite is produced and leads to glutathione depletion and free radical formation (3). It has been well confirmed that during liver toxicity induced by APAP, apoptosis and necrosis result from oxidant-antioxidant imbalance (4-7). During the 1960s and 1970s, the first cases of APAP toxicity were reported due to its excessive administration in the world, so that it became a global problem (8, 9). According to statistics, the administration of more than 4g/day of APAP among the adult population of the United States of America has been reported that about 30000 cases were admitted in hospital (10). Therefore, the treatment of acetaminophen poisoning must be a lot of attention. Currently, N-acetyl-cysteine is used as an antidote for hepatotoxicity of APAP. Intravenous NAC is associated dose dependent adverse effects, which can lead to treatment interruption (11). However, modified NAC regimen has led to a reduction in vomiting, and anaphylactoid reactions (12). It has been reported that the current dose of NAC is not sufficient to treat APAP poisoning in higher plasma concentrations of APAP, despite prompt treatment (13). Another strategy to protect liver against APAP toxicity is induction of autophagy by pharmacologic agents. Autophagy protects hepatocytes against APAP toxicity by removing damaged mitochondria and APAP protein adducts (14, 15). Doxycycline (DC) is a tetracycline-derived antibiotic and is mainly used for pulmonary infections and skin and kidney problems (16). Interest in DC is because of antioxidant and anti-inflammatory effects and inhibitory activity on matrix metalloproteinase (MMP), the activities distinct from the antibacterial action. Based on previous studies, DC inhibits metalloproteinase and in this way affects inflammation, neoplasia, and fibrosis (17-19). The antioxidant and anti-inflammatory properties of DC can be important for management and control of cellular damages (20). The therapeutic efficacy of DC has been shown in resolving gingivitis and periodontitis (21). Therapeutic agents that are presented as a hepatoprotective should not cause liver damage. Regarding the association between DC and hepatotoxicity, it has been reported that DC users did not show an increased risk of hepatotoxicity and DC could be a safe tetracycline-derived antibiotic (22). NAC complications lead to discontinuation of the drug during the treatment of APAP poisoning. Furthermore, NAC side effects increase by increasing its dose, thus the search for other treatments should be considered. Given that above studies, probably DC could be a good candidate for treatment liver toxicity induced by APAP. Therefore, the aim of this study was to assess the effects of DC on APAP-induced acute liver toxicity in mice.

## Experimental

*Animals*

In this study, seventy-two male BALB/c mice weighing 25 ± 2 g were purchased from the Animal Center of Ahvaz Jundishapur University of Medical Science (Ahvaz, Iran). They were housed in room temperature (25 ± 2 ºC) and 12/12 h light-dark cycle and given standard rat chow and drinking water ad libitum. The mice are fasted overnight to deplete glutathione (GSH) stores in order to better induce APAP toxicity (23). Animal care, use, and ethical issues were based on Ethical Committee Acts of Ahvaz Jundishapur University of Medical Sciences (AJUMS) for care and use of laboratory animals (IR.AJUMS.REC.1395.40).

*Experimental procedure*

In this study, the effects of different doses of DC on biochemical and histopathological parameters of acute APAP hepatotoxicity were examined in two 3 h and 24 h time periods. For this purpose, the animals were divided into six groups (n = 12, six in each time).

Group I (control), the animals were treated by normal saline; Group II (APAP), the animals were treated by 400 mg/kg APAP intraperitoneally (i.p.); Group III-V, DC co-administered with APAP at doses of 25, 50, and 100 mg/kg and Group VI (DC 100) received DC 100 mg/kg. The used doses of DC are based on the previous studies (24, 25). Doxycycline was administered i.p. in a single dose just before APAP. Finally, the mice were euthanized by cervical dislocation under mild anesthesia in two periods of 3 h and 24 h and blood sampling performed by the jugular vein so that six animals in each period were examined. The serum was instantly separated using centrifuge (3500 RPM, 15 min) for biochemichal assessment. After blood sampling, the liver was removed and one part was considered for hepatic tissue markers and another was fixed in 10% formalin for histopathological examination.

*Evaluation of serum liver biomarkers*

To evaluate the ALT and AST activity, serum samples from the animals were tested based on commercial kit protocol (Pars Azmoon, Tehran, Iran) through autoanalyzer (Biotechnical BT-3000 plus Chemistry Analyzer, Italy).

*Liver homogenization*

At first, liver was homogenized in phosphate buffer. The obtained suspension was centrifuged (6000 RPM, 10 min) and supernatant was removed for subsequent evaluation.

*Catalase activity evaluation in liver*

For assessment the activity of catalase in liver, 50 µL supernatant was added to 950 µL buffer containing hydrogen peroxide 20 mM. In the next stage, the absorbance amount was read instantly and after 1 min at 240 nm, respectively. Then the catalase activity level was reported as µmol H_2_O_2_/min/g tissue (26).

*Reduced GSH assessment in liver*

The reduced GSH level is measured by Ellman’s reagent (27). In brief, 20% trichloroacetic acid (TCA) with 1 mM EDTA was added to homogenized liver. In next stage, it was centrifuged (10 min, 200 rpm) and isolated supernatant (200 µL) was added to 0.1mM Ellman’s reagent (5, 5′-dithio bis-2-nitrobenzoic acid) (1.8 mL). The absorbance amount was read at 412 nm by spectrophotometer and GSH level was reported as µmol/g tissue.

*Measurement of lipid peroxidation in liver*

Lipid peroxidation in the liver is determined based on the reaction between MDA and thiobarbituric acid (TBA), which produces a purple color with maximum absorbance at 532 nm (28). Thereafter, 1 mL supernatant was added to 2 mL TBA and placed in 100 cº for 15 min. After cooling, it was centrifuged (3000 RMP, 10 min) to separate the organic layer. Finally, the absorbance amount was read at 532 nm and MDA level was reported as nmol/g tissue.

*Histopathological examination*

For evaluation of microscopic changes, the liver was fixed at 10% formalin. Then, it was dehydrated by soaking in alcohol and xylol, respectively. Finally, after preparation of 5µ-tissue sections using a rotary microtome, the haematoxylin and eosin (H&E) staining was performed. The histopathological changes including degeneration of hepatocytes, fatty change in hepatocytes, coagulative necrosis in hepatocytes, infiltration of inflammatory cells, and dilated sinusoids were examined using a light microscope.

*Statistical analysis*

All statistical analyses were carried out using the Prism 5.0 (San Diego, CA, USA) statistical package program. The variables are expressed as mean ± SD. Comparison of mean values was performed by one-way ANOVA followed by Tukey’s *post-hoc* test. Significance was set at *P* < 0.05.

## Results

*Hepatic index*

**Figure 1:**
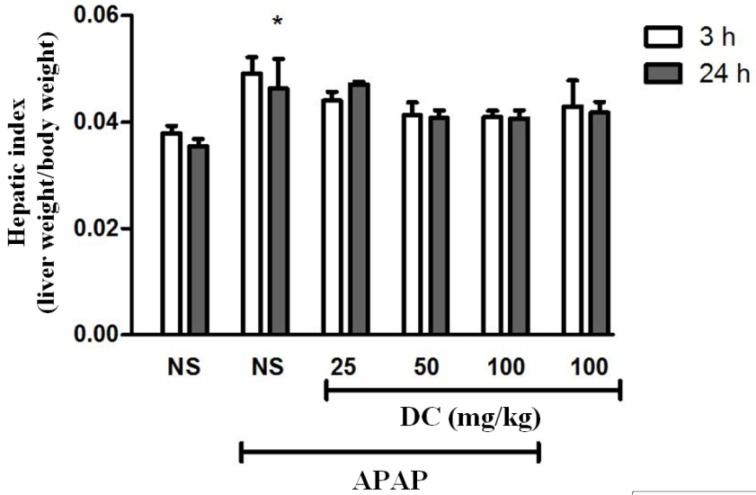
Effect of doxycycline (DC) on hepatic index. The animals were treated with DC (25, 50 and 100 mg/kg, i.p.) or normal saline (NS) just before APAP 400 mg/kg

As shown in Figure 1, the hepatic index (as the ratio of wet liver weight to body weight) significantly increased 24 h after administration of APAP compared to normal saline control group (*P* < 0.05). However, in all groups treated with DC there was no significant change in the level of hepatic index in comparison with APAP group.

*Serum liver biomarkers*

**Figure 2:**
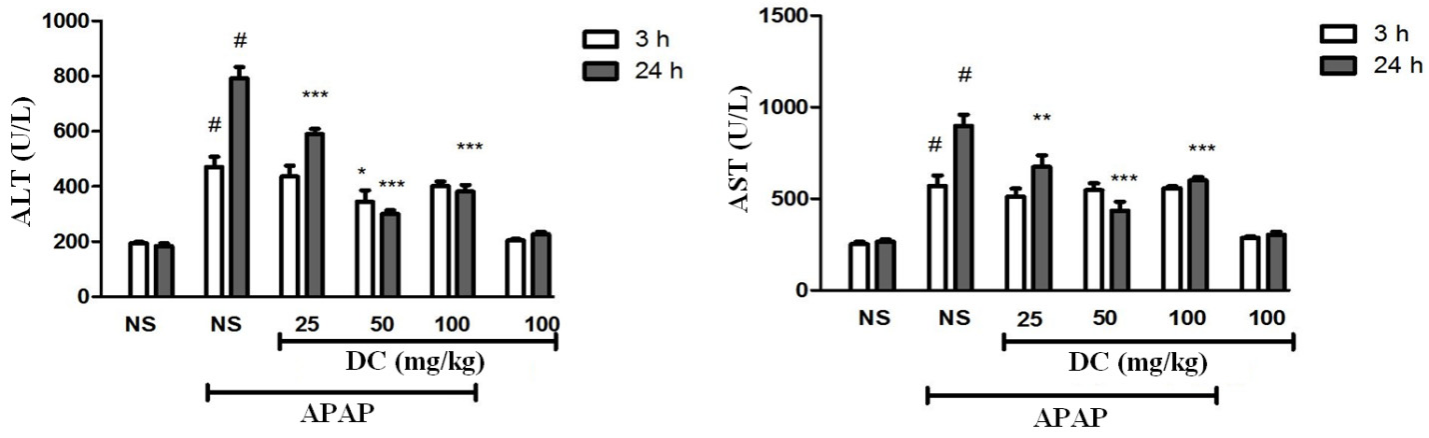
Effects of doxycycline (DC) on serum activity of ALT and AST. The animals were treated with DC (25, 50 and 100 mg/kg, i.p.) or normal saline (NS) just before APAP 400 mg/kg

The evaluation of serum AST and ALT biomarkers indicated that DC at the all doses could normalize their levels to normal range over a 24 h period (Figure 2). In 3 h-period treatment, a significant reduction in serum level of ALT was observed in groups treated by 50 mg/kg DC compared to saline control group (*P* < 0.05), while the results obtained from groups treated with 25 and 100 mg/kg DC were not significant. Nevertheless, the results confirmed that DC (all doses) led to significant reduction of serum liver biomarkers at the end of 24 h time period, so that decreasing effects in dose of 50 mg/kg DC were higher than doses of 25 and 100 mg/kg DC.

*The evaluation of antioxidant conditions*

**Figure 3:**
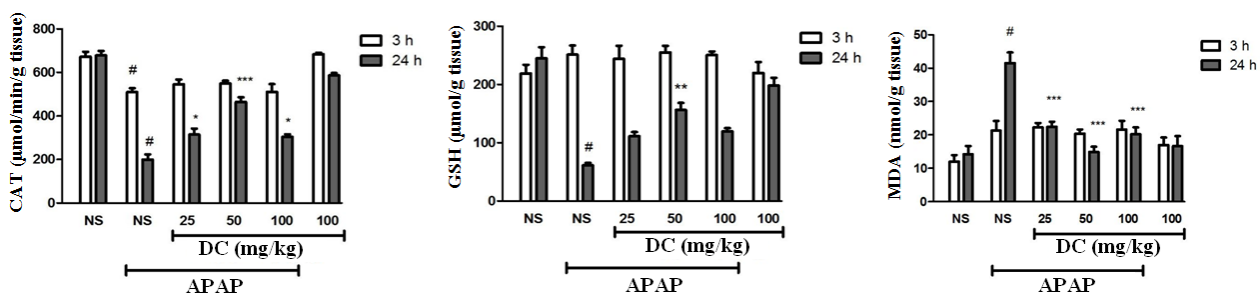
Effects of doxycycline (DC) on the activity of catalase, GSH and MDA levels in the liver. The animals were treated with DC (25, 50 and 100 mg/kg, i.p.) or normal saline (NS) just before APAP 400 mg/kg

Our findings indicated that APAP is a main factor in reducing catalase activity in the liver so that administration of 400 mg/kg APAP leads to dramatic reduction of catalase activity in both periods. In addition, we confirmed that DC enhances the reduced activity level of catalase at the end of 24 h treatment period (Figure 3). APAP leads to a dramatic reduction in GSH levels in the liver at the end of 24 h-period. The results confirm beneficial effect of DC in normalization of glutathione level especially in dose of 50 mg/kg at the end of 24 h period. However, glutathione levels were increased in APAP groups treated with DC 25 and 100, but this elevation was not significant in 24 h time duration (Figure 3). Evaluation of MDA as a major index of lipid peroxidation confirms that induction of hepatic toxicity by APAP results in an increase of malondialdehyde level in the liver. Indeed, lipid peroxidation is a common event during APAP-induced liver toxicity. Treatment with DC at all doses could decrease the MDA level at the end of 24 h period (Figure 3).

*Histopathological findings*

**Figure 4:**
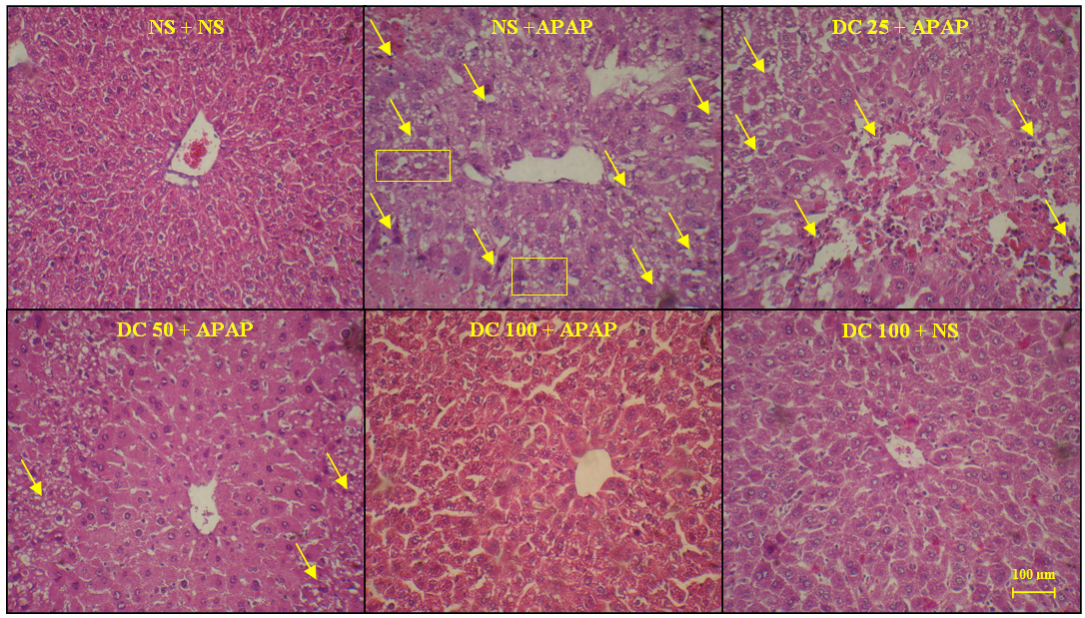
The liver sections regarding protective effects of doxycycline (DC) on hepatotoxicity of APAP in mice 3h after APAP. The animals were treated with DC 25, 50 and 100 mg/kg or normal saline (NS) just before APAP 400 mg/kg (H&E x300)

**Figure 5:**
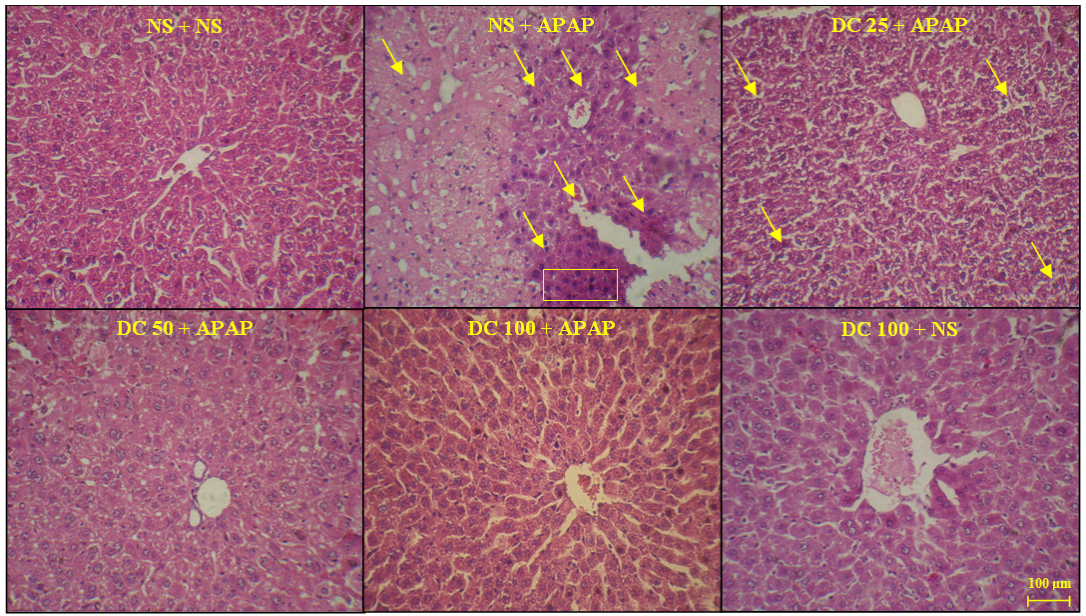
The liver sections about protective effects of doxycycline (DC) on hepatotoxicity of APAP in mice 24 h after APAP. The animals were treated with DC 25, 50 and 100 mg/kg or normal saline (NS) just before APAP 400 mg/kg (H&E x300)

As shown in Figures 4 and 5, the liver structure in group received DC 100 mg/kg was similar to that of the group treated with normal saline and any pathological changes were not observed at the end of 3 h and 24 h periods of treatment. This indicates that in this study the high dose of DC (100 mg/kg) is causing no damage and is practically safe. The results also confirmed that administration of APAP leads to damages such as the lack of radial arrangement, the destroying of sinusoids, the presence of eosinophils, and several necrotic hepatocyte followed by a 3 h and 24 h periods. At the end of 24 h period, the pyknotic nuclei were also seen. The photomicrographs examination of animal groups showed that hepatoprotective effects of DC are dose-dependent so that by increasing the DC dose, liver tissue parameters have been improved.

## Discussion

The main objective of our study was to investigate the protective effect of DC on APAP-induced liver injury in mice. The liver potential for detoxification of drugs is well known (29). If APAP administered in therapeutic doses, there will be no particular side effects in the liver, but it can be a serious threat for liver during overdose (30). Indeed, its overdose is one of the main causes of liver injury through the production of NAPQI as a toxic substance by cytochrome P450 activation and ultimately reactive oxygen species formation (31). We found that administration of APAP (400 mg/kg) leads to significant changes in serum biomarkers, antioxidant status as well as histopathology parameters in mice. However, these changes are more evident at 24h period than 3h. These findings are analogous to previous studies (29, 32, 33). Our results showed that hepatic index had not apparent change subsequently treatment with DC.

*Significantly different from control normal saline group in 24 h period study (*P *< 0.05).

**#**(*P *< 0.001) significantly different from control normal saline group in both times.

*(*P *< 0.05), **(*P *< 0.01) and ***(*P*0.001 < ) significantly different from APAP treated mice.

**#**(*P *< 0.05) significantly different from control normal saline group in both times.

*(*P *< 0.05), **(*P *< 0.01) and ***(*P *< 0.001) significantly different from APAP treated mice.

**Figure 6:**
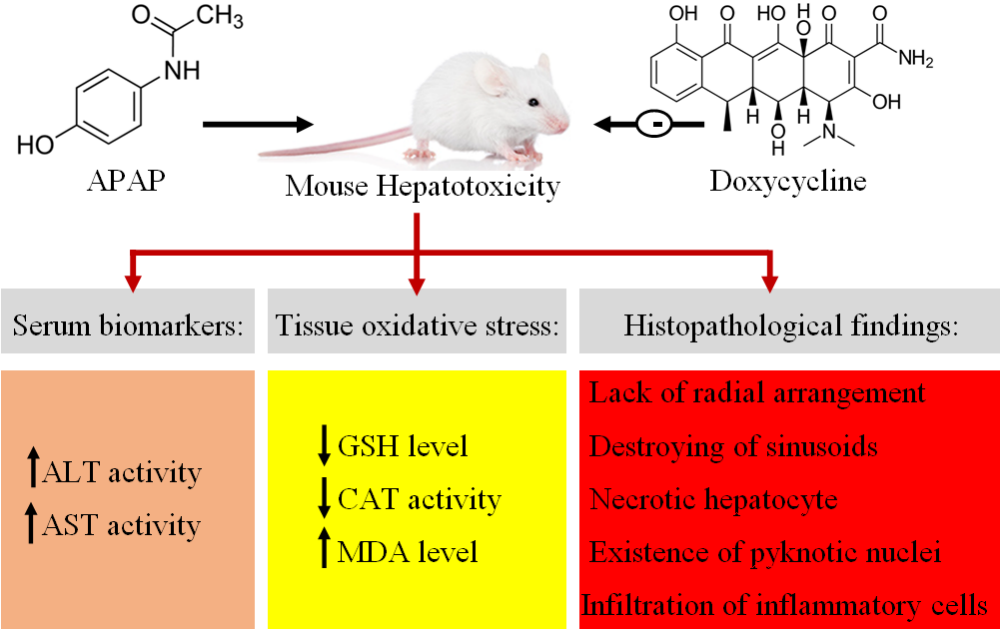
Graphical abstract regarding protective effects of doxycycline on acute APAP induced liver toxicity in mice

Any increase in ALT and AST activity indicates hepatic injury (34). Evaluation of serum aminotransferases activity level confirmed that DC results in the normalization of liver biomarkers since DC significantly reduced the activity level of ALT and AST at the end of the experiment.

In cells, the protection against free radicals is performed by antioxidant systems (35). During liver toxicity induced by APAP, the abnormality of catalase activity and glutathione content occur in the liver due to free radicals formation (3, 32, 36-38). In addition, one of the events to oxidative stress under APAP toxicity is lipid peroxidation followed by MDA increase (35). Our findings revealed that DC has antioxidant effect on liver toxicity induced by APAP. Indeed, DC could improve the level of MDA and GSH as well as activity of catalase to normal range and the best results were observed to the dose of 50 mg/kg of DC.

It has been demonstrated that APAP administration results in massive changes in liver structure in the mice. It is well known that the mice are a human-relevant model of APAP hepatotoxicity. Mice are much more sensitive than rats and rats develop little or no liver injury (31, 39). In conjunction with histopathological findings, the results indicated that treatment with 100 mg/kg DC leads to a clear decrease in APAP induced hepatic injury. In addition, dose of 50 mg/kg could partially reduce these injuries.

Fujita *et al*, indicated that DC has anti-inflammatory and antifibrotic properties agianst pulmonary injury induced by bleomycin due to its antioxidant property (19). It has been reported that DC has neuroprotective effects in pilocarpine model of convulsion in rats by antioxidant and the radical scavenging effects (25). Furthermore, antioxidant properties of DC lead to improvement of injury induced by doxorubicin in heart (40), ischemia/reperfusion in liver (41) acute ischemia/reperfusion (42) and hypoxia-reoxygenation in the kidney (20). In confirmation of the mentioned studies, we also found that DC has antioxidant properties. Based on study conducted by Kholmukhamedov *et al*., administration of DC improves damage induced by hemorrhagic shock/resuscitation in the liver and kidney throug abrogation of apoptosis and necrosis (43). In this study, DC decreased infiltration of inflammatory cells to liver tissue. In confirmation, anti-inflammatory activities of DC have been shown in experimental models of nociception and inflammation (24, 44). The group treated by 50 mg/kg DC had more improvement in serum and liver biochemical markers, therefor, it may consider that it is most affective dose in improvement of liver damage induced by APAP. Furthermore, based on the histopathological examination it seems that the dose of 100 mg/kg is the most effective dose. Nevertheless, in a case report study has been demonstrated that administration of bupropion and DC lead to jaundice and malaise in an African American patient (45). Matrix metalloproteinases have been involved in acute and chronic liver injury and MMP-9 as an important agent in recruitment of leukocytes can be a good target in resolving acute liver injury (46). It has been reported that DC improves gap junction remodeling by inhibiting MMP activity in rat model ligation of the left anterior descending branch of the coronary artery (47). However, inhibitor activity of minocycline on MMP-9 is more than DC and tetracycline (48). Thus, DC and other tetracyclines may be useful in preventing and resolving liver disorders. 

## Conclusion

Finally, APAP as one of the major causes of hepatotoxicity leads to oxidant-antioxidant imbalance and subsequently increases the activity of serum biomarkers and liver tissue oxidative damages with infiltration of inflammatory cells (Figure 6). APAP toxicity reaches maximum after 24 h and 3 h is not enough to cause prominent damage. Overall, it can be concluded that DC protect liver against APAP toxicity by antioxidant and anti-inflammatory effects.

## Competing Interests

The authors declare no financial conflicts of interest for this study.
